# Effect of Intraperitoneal Etanercept on Oxidative Stress in Rats with Peritonitis

**DOI:** 10.1155/2016/9418468

**Published:** 2016-03-14

**Authors:** Yasar Yildirim, Esma Gulsum Cellad, Ali Veysel Kara, Zülfükar Yilmaz, Ali Kemal Kadiroglu, Mehmet Veysi Bahadir, Mesut Gul, Muzaffer Aydin Ketani, Mehmet Emin Yilmaz

**Affiliations:** ^1^Department of Nephology, Dicle University Faculty of Medicine, 21090 Diyarbakir, Turkey; ^2^Department of Internal Medicine, Dicle University Faculty of Medicine, 21090 Diyarbakir, Turkey; ^3^Department of General Surgery, Dicle University Faculty of Medicine, 21090 Diyarbakir, Turkey; ^4^Department of Histology and Embryology, Dicle University Faculty of Veterinary Medicine, Turkey

## Abstract

Our aim was to evaluate effect of etanercept on oxidative stress parameters in rats with experimental peritonitis and investigate the availability of etanercept usage in the treatment of peritonitis in the future. Twenty-eight rats were divided into four groups as control (group 1), peritonitis (group 2), peritonitis + cefazolin sodium (group 3), and peritonitis + cefazolin sodium + etanercept (group 4). Peritoneal tissue and blood samples were taken from all of the rats for histopathological and biochemical examination. The oxidative stress parameters were examined in blood and tissue samples. It was observed that rats with peritonitis benefit from cefazolin sodium treatment. Evaluating the effectiveness of etanercept was our main objective for this study. In this perspective, we compared group 3 and group 4 and found statistically significant decreases in oxidative parameters and statistically significant increases in antioxidants in serum and tissue samples in group 4. It is observed that there was a significant contribution of etanercept on biochemical and also histopathological results. As a result, the TNF-*α* inhibitor, etanercept, in addition to antibiotics given in the early treatment of peritonitis results in more significant improvement of histopathological and oxidative parameters as compared to antibiotics alone.

## 1. Introduction

Peritonitis, which is the inflammation of the peritoneal tissue, can cause systemic inflammatory response and sepsis. This most likely occurs because infectious agents can pass rapidly from the peritoneal surface to circulation [1]. Peritoneal sepsis is a clinical entity with high rates of morbidity and mortality. Therefore, limiting peritonitis in early stages is of paramount importance. In fact, proinflammatory cytokines such as TNF-*α* and IL-1 are increased as a part of the inflammatory response in order to limit tissue damage [2]. However, overproduction of proinflammatory cytokines disrupts the normal immune response and causes a pathological response. As a result, capillary leakage, tissue damage, and multiorgan failure can occur. TNF-*α* plays an important role in this process and is considered as the main mediator in the early stage of inflammation [3]. Etanercept is the competitive inhibitor of TNF-*α* which inhibits the binding of TNF-*α* to cell surface receptors and limits its biological activity. Suppression of TNF-*α* by etanercept in the case of excessive immune response can play an important role in the limiting of inflammation [4]. Inflammation causes oxidative stress which often results in decreased antioxidant levels and increased production of oxidant. The presence of oxidative stress is an important parameter which can be measured to indicate tissue damage.

## 2. Material and Methods

Our experimental study was conducted in Dicle University by Professor Dr. Sabahattin Payzın at the Health Science Research and Application Center (DUSAM) with the approval of ethical committee for animal experiments. The project was supported by Dicle University Coordination of Scientific Research Projects (project number 13-TF-34). A total of 28 adult female Wistar Albino rats weighing between 200 and 250 g were used and fed with standard chow and water prior to the experiment.

Twenty-eight rats were divided into four groups (*n* = 7 per group) as follows:

 Group 1: control group, in which rats did not receive any drugs.
 Group 2: peritonitis group, in which intraperitoneal injection of 1.5 mL* Escherichia coli* suspension (107 CFU/mL) was made to cause experimental peritonitis.
 Group 3: peritonitis + cephazolin sodium group, in which intraperitoneal cephazolin sodium injection (50 mg/kg) was made one hour after the injection of* E. coli* suspension.
 Group 4: peritonitis + cephazolin sodium + etanercept group, in which intraperitoneal cephazolin sodium injection was made one hour after the injection of* E. coli* suspension (50 mg/kg). Also intraperitoneal etanercept injection was made one and four hours after the injection of* E. coli* suspension. The study was terminated 24 hours after the etanercept injection.

Ketamine hydrochloride (70 mg/kg) was administered intramuscularly (im) to rats before surgery. Rats under anesthesia were fixed at the supine position. Midline incision of the anterior abdominal wall was performed on all of the rats for histopathological and biochemical examination. Full thickness tissues except skin with 1 cm of length and 3 mm of thickness were taken from left half and right half of the midline abdominal wall, respectively, for biochemical and histopathological examination. Tissue samples for histopathological examination were fixed in 10.0% buffered formalin. Tissue samples for biochemical examination were taken into the aluminum foil. Then, sternotomy was made and 5 mL blood sample was taken from the heart. Also, with this method, killing of the animals was achieved.

### 2.1. Biochemical Analysis

Blood was centrifuged at 5,000 rpm and 4°C for 8 minutes and supernatants were collected for study. Tissue samples were placed in refrigerator at −20°C until homogenized. Tissue homogenization was performed using a homogenizer in the laboratory. After that, paraoxonase (PON), malondialdehyde (MDA), nitric oxide (NO), total antioxidant capacity (TAC), total oxidant stress (TOS), and tumor necrosis factor alpha (TNF-*α*) were studied from tissue and blood samples in the biochemical laboratory of Dicle University.

PON was studied in the Abbott Architect® c16000 autoanalyzer by using RL0031 Rel Assay® Diagnostics Paraoxonase (Gaziantep, turkey) kit. NWLSS (Northwest Life Science Specialties) Malondialdehyde Assay kit was used for malondialdehyde and reading was made at 450 nm by Dynex micro ELISA device. Cayman Chemical Company 780001 Nitrite Colorimetric Assay kit was used for NO and reading was made at between 540 nm and 550 nm by Dynex micro ELISA device. TAC was studied in in the Abbott Architect® c16000 autoanalyzer by using RL0017Rel Assay® Diagnostics TAS kit (Gaziantep, Turkey). TOS was studied in the Abbott Architect® c16000 autoanalyzer by using RL0024 Rel Assay® Diagnostics (Gaziantep, Turkey) TOS Assay kit. TAC AND TOS were studied by the method of Erel. Serum levels of TNF-*α* were measured by Biosource Rat TNF-*α* kit (lot number KRC3011), which is a solid phase sandwich enzyme linked immune sorbent assay (ELISA).

### 2.2. Histopathological Analysis

Paraffin sections of 4-5-micrometer thickness were taken from tissue samples by using rotary microtome. These sections were stained with hematoxylin and eosin (H&E) and examined by the Nikon Eclipse 400 digital camera (Nikon DSRi). Peritoneal epithelial shedding (desquamation), congestion in the lamina propria, neutrophil infiltration in the lamina propria, and edema in the lamina propria were evaluated histopathologically and scored from 0 to 4 (Table 1).

### 2.3. Statistical Analysis

Data analyses were performed using Statistical Package for Social Sciences (SPSS), Version 16.0 for Windows. All the data are presented as mean ± standard error. Kruskal-Wallis test was used to analyze multiple groups and Mann-Whitney *U* test was used for binary comparison of groups. *p* < 0.05 was considered statistically significant.

## 3. Results

We found statistically significant differences between group 1 and group 2 in terms of oxidative parameters and antioxidants in the serum and peritoneal tissue. There were an increase in oxidative parameters and a decrease in antioxidants in group 2 according to group 1. When we compared group 2 and group 3, there was a statistically significant decrease in the oxidative parameters (MDA, TOS, and TNF-*α*) in serum and tissue samples in group 2, whereas there was a statistically significant increase in the antioxidant parameters (TAC, PON) in serum and tissue sample in group 3 (Table 2). However, there was no statistically significant difference between groups in terms of NO in serum (*p* = 0.090) and the tissue samples (*p* = 0.264). Evaluating the effectiveness of etanercept was our main aim in this study. In this perspective, we compared group 3 and group 4 and found statistically significant decreases in oxidative parameters (MDA, TNF-*α*, TOS, and NO) in serum and tissue samples and statistically significant increases in antioxidants (TAC, PON) in serum and tissue samples in group 4 (Table 2). These results were more prominent in comparison of group 2 and group 4.

**Figure 1:**
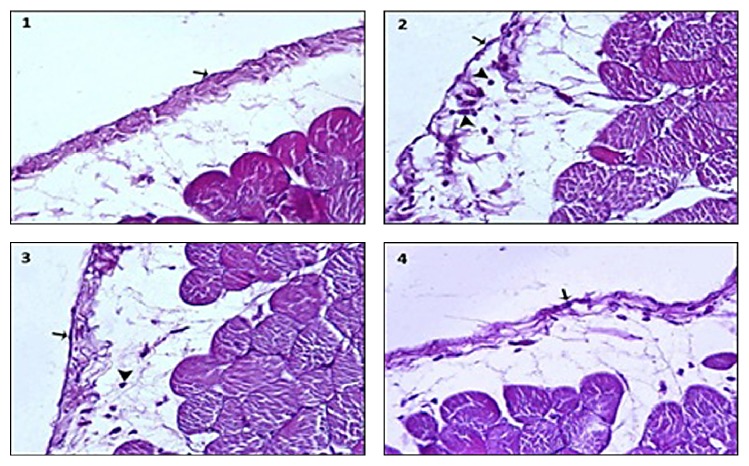
Microscopic findings of the parietal peritoneum in groups ((1) control group, (2) peritonitis group, (3) peritonitis + cephazolin sodium group, and (4) peritonitis + cephazolin sodium + etanercept group).

Microscopic findings of parietal peritonitis in the groups were shown in Figure 1. In our study, we also found that there were no histopathological changes in peritoneal epithelium and lamina propria in group 1. In group 2, there were peritoneal epithelial shedding, diffuse neutrophil infiltration and edema in the lamina propria, and congestion in capillary vessels. We observed that there was a statistically significant reduction in peritoneal epithelium shedding (*p* < 0.01), edema (*p* < 0.01), congestion, and neutrophil infiltration in the lamina propria (*p* < 0.01) in group 3 compared to group 2 due to effect of cephazolin sodium (Table 3). In group 4, in which we used etanercept, there was a statistically significant reduction in the epithelial shedding, congestion, edema, and neutrophil infiltration in the lamina propria according to both group 2 (resp., *p* < 0.01, *p* < 0.01, and *p* < 0.001) and group 3 (resp., *p* < 0.01, *p* < 0.05, and *p* < 0.01). There were statistically significant differences between groups in terms of histopathological results (Table 3).

## 4. Discussion

Peritonitis is the inflammation of visceral or parietal peritoneum. It can cause systemic inflammatory response and sepsis if not treated properly. It is still one of the significant causes of morbidity and mortality worldwide despite improvements of treatment methods, intensive care unit conditions, and intensive care units devices. Peritonitis is not a simple infection process; inflammation and immunological dysregulation are activated by different mechanisms and can lead to multiple organ failure. Therefore, peritonitis is an important clinical table, which can cause multiple organ failure. Limiting this process in stage of peritonitis is crucial. It is thought that there are some unexplained pathologic mechanisms in this process and therefore experimental models are needed.

Peritoneal membrane is semipermeable and has an advanced ability of secretion and absorption. If urea, fluids with electrolytes, drugs, and infective material are given to peritoneal cavity, they can rapidly pass to systemic circulation. Therefore, peritonitis models in experimental animal studies are valuable for creating sepsis and they are closest designs to human sepsis.

Inflammation is an important part in the formation of sepsis. Proinflammatory cytokines such as TNF-*α* and IL-1 were secreted to limit tissue damage. However, overproduction of proinflammatory cytokines disturbs the normal order of immune response and can lead to a pathological inflammatory response. This situation results in capillary leakage, tissue damage, and multiple organ failure [3]. TNF-*α* plays an important role in the inflammation process and is considered a master mediator of early stage inflammation. Guo et al. found that TNF-*α* levels were highest in the early period after injection (30 min–1 h) in a sepsis model elicited lipopolysaccharide injection [5]. Etanercept is the competitive inhibitor of TNF-*α*, which inhibits the binding of TNF-*α* to cell surface receptors and limits its biological activity. Suppression of TNF-*α* by etanercept in the case of excessive immune response can play an important role in the limiting of inflammation. Karabacak and Yazar predicted clinical benefit [6] and surveillance may also improve [5].

Release of oxygen particles due to infectious and immunological causes can alter the lipid, protein, and DNA structure of cells and results in cell damage and death [7, 8]. In addition to this, antioxidants are also released to protect against harmful effects of oxidants. Stable and sufficient function of this system is important to deal with harmful effects of oxidative stress [9].

According to these pieces of information, we created an experimental peritonitis model in the rats, and we injected intraperitoneal etanercept in early stage to suppress inflammation. We aimed to show the effects of intraperitoneal etanercept on oxidant and antioxidant parameters. Therefore, we also indirectly investigated effect of etanercept on preventing oxidative damage in sepsis and peritonitis clinics. We created peritonitis in the rats by injection of 1.5 mL (107 CFU/mL)* E. coli* suspension, followed by dividing them into 4 groups: group 1, control group; group 2, peritonitis group; group 3, peritonitis + cephazolin sodium group; group 4, peritonitis + cephazolin sodium + etanercept group.

Antibiotics play a main role in the treatment of infectious peritonitis. Therefore, many researchers have compared the effectiveness of various antibiotics in peritonitis [10, 11]. In our study, the use of cephazolin sodium as effective antibiotic in the treatment of peritonitis was preferred [10]. Intraperitoneal cephazolin sodium in a dose of 50 mg/kg was given to groups 3 and 4 one hour after the injection of* E. coli* suspension. There are many clinical studies made by antibiotics or antibiotics and other drugs combinations to limit peritonitis such as antibiotic + vitamin E [12], antibiotics + normobaric oxygen therapy [13], and antibiotics + oxygen-free radicals scavengers [14]. It can be seen that etanercept has been used for suppression of inflammation in autoimmune diseases such as rheumatoid arthritis [15], ankylosing spondylitis [16], psoriatic arthritis [17], and psoriasis vulgaris [18]. We also observed that etanercept has been used experimentally in the nonautoimmune disease. It was shown that etanercept can improve neuroinflammation and myocardial ischemia reperfusion injury [19, 20].

It is known that TNF-*α* is responsible for early responses of inflammation, which was secreted minutes after the inflammatory stimulus, peaked at first hour, and stopped after 3-4 h [21]. Therefore, we injected etanercept at first hour and TNF-*α* peaked at the 4th hour when secretion of TNF-*α* stopped. We injected etanercept in a dose of 8 mg/kg which is not toxic but can block TNF-*α* [22, 23]. We basically compared these two treatment methods by looking to serum and tissue levels of MDA, NO, TNF-*α*, TOS, TAC, and PON. We evaluated the histopathological findings in all groups. An increase in levels of MDA, TNF-*α*, and TOS shows oxidative stress and an increase in levels of TAC and PON shows antioxidant activity. Upon a literature search, we found that NO has been shown to be cytoprotective at low levels and cytotoxic at high levels unlike the other parameters [24].

If we correlate the histopathological findings with the oxidant and antioxidant parameters variability in serum and tissue samples, we can conclude that addition of etanercept to cephazolin sodium treatment in early stages of peritonitis treatment can be useful for limiting the peritonitis. Increased suppression of TNF-*α* levels with the usage of etanercept seems to be useful for limiting peritonitis. In a study by Chen et al., they found decreased antioxidant levels in rats with subacute peritonitis compared with the control group [25]. Similar to this study, we also found decreased antioxidant levels. Di Paola et al. showed an increase in TNF-*α* levels in infected tissues of rats with periodontitis compared with control group [26]. We also found an increase in TNF-*α* levels in groups with peritonitis compared with control group (TNF-*α* levels in group 1: 116.39 ± 42.36 pg/mL and TNF-*α* levels in group 2: 487.21 ± 238.44 pg/mL, *p* = 0.002). A decrease in TNF-*α* levels by using etanercept in the same study was also seen in group 4 in our study (TNF-*α* levels in group 4: 136.83 ± 24.26). We also showed a statistically significant decrease in MDA levels in serum (*p* < 0.001) and tissue samples (*p* < 0.001) in group 4 compared with group 2 similar to decrease in MDA levels by using etanercept in the study [26]. A decrease in TNF-*α* and MDA by using etanercept was collocated with histopathological improvement. We found increased NO levels in peritonitis group similar to previous reports [27]. In our study, there were statistically significant differences between group 1 and group 2 in terms of NO levels in serum (*p* = 0.001) and tissue samples (*p* = 0.001). Unlike other parameters, there was a statistically nonsignificant decrease in NO levels in group 3. In group 4, there were statistically significant decreases in NO levels. PON, which is a powerful antioxidant, decreases in case of infection [28, 29]. There was a statistically significant difference between group 1 and group 2 in terms of serum (*p* = 0.001) and tissue (*p* < 0.001) for PON levels. PON levels were decreased in peritoneal tissue (1.83 ± 2.54 U/L) and serum (124.61 ± 15.81 U/L) in rats with peritonitis. With the addition of the treatment, there was an increase in PON levels in serum and tissue samples in group 3 and group 4. This increase was prominent in group 4 in terms of both serum (*p* = 0.029) and tissue samples (*p* < 0.001).

Consistent with the literature, there was a decrease in oxidative parameters and an increase in antioxidant levels by using etanercept in addition to cephazolin sodium in rats with experimental peritonitis. A decrease in the edema, congestion, and neutrophil infiltration in the lamina propria of the peritoneum was another important finding of this study. It appears that the suppression of TNF-*α* in early stage can reduce excessive immune response resulting in the destruction of the organism.

As a result, etanercept, in addition to antibiotics given in the early treatment of peritonitis, results in more significant improvement in histopathological and oxidative parameters according to antibiotics alone. We believe that etanercept can decrease sepsis incidence in peritonitis but there is a need for more comprehensive experimental studies for usage in the treatment of peritonitis.

## Figures and Tables

**Table 1:** Histopathological scoring system for the tissue evaluation.

Absent	Minimal	Weak	Moderate	Strong
0	1	2	3	4

**Table 2:** Comparison of serum and tissue oxidant and antioxidant parameters.

Parameters	Group 1	Group 2	Group 3	Group 4
	(*n* = 7)	(*n* = 7)	(*n* = 7)	(*n* = 7)
Serum				
MDA (mmol/mL)	0.73 ± 0.17	1.67 ± 0.32^d^	1.28 ± 0.21^b^	0.90 ± 0.26^c,e^
TOS (*μ*mol/L)	33.59 ± 11.23	80.30 ± 11.26^d^	52.73 ± 12.48^c^	39.07 ± 7.07^c,e^
NO (*μ*M/L)	4.85 ± 0.39	5.80 ± 0.46^a^	5.41 ± 0.32^c^	4.90 ± 0.32^b,c^
TNF-*α* (pg/mL)	2.72 ± 1.45	18.50 ± 10.01^a^	9.01 ± 2.06^b^	5.77 ± 2.34^b,c^
TAC (mmol/L)	1.32 ± 0.10	0.87 ± 0.30^a^	1.18 ± 0.10^b^	1.56 ± 0.11^e,f^
PON (U/L)	197.03 ± 37.63	124.61 ± 15.81^a^	145.67 ± 7.75^b^	157.70 ± 10.25^b,c^
Peritoneal tissue				
MDA (mmol/mL)	0.12 ± 0.25	1.31 ± 0.41^d^	0.59 ± 0.35^b^	0.12 ± 0.07^c,e^
TOS (*μ*mol/L)	7.16 ± 3.53	45.90 ± 26.57^a^	14.18 ± 4.58^b^	8.77 ± 4.11^b,c^
NO (*μ*M/L)	3.95 ± 0.87	6.65 ± 1.36^a^	6.00 ± 0.52	5.29 ± 0.22^b,c^
TNF-*α* (pg/mL)	116.39 ± 42.36	487.21 ± 238.44^a^	265.22 ± 52.74^b^	136.83 ± 24.26^b,f^
TAC (mmol/L)	0.80 ± 0.11	0.34 ± 0.08^d^	0.47 ± 0.064^b^	0.66 ± 0.14^c,e^
PON (U/L)	12.74 ± 3.32	1.83 ± 2.54^d^	4.82 ± 1.37^b^	8.34 ± 1.14^e,f^

MDA: malondialdehyde, NO: nitric oxide, TNF-*α*: tumor necrosis factor alpha, TAC: total antioxidant capacity, TOS: total oxidant stress, and PON: paraoxonase.

^a^ *p* < 0.005 as compared to group 1, ^b^ *p* < 0.005 as compared to group 2, ^c^ *p* < 0.005 as compared to group 3, ^d^ *p* < 0.001 as compared to group 1, ^e^ *p* < 0.001 as compared to group 2, and ^f^ *p* < 0.001 as compared to group 3.

**Table 3:** Histopathological results.

Histopathological results	Control group (group 1)	Peritonitis group (group 2)	Peritonitis + cephazolin sodium group (group 3)	Peritonitis + cephazolin sodium + etanercept group (group 4)
Peritoneal epithelial shedding (desquamation)	0.0 ± 0.0	3.71 ± 0.48^a^	2.71 ± 0.48^c^	1.0 ± 0.57^c.e^
Congestion	0.0 ± 0.0	4.0 ± 0.0^b^	2.42 ± 0.78^c^	1.28 ± 0.48^c.f^
Neutrophil infiltration	0.0 ± 0.0	4.0 ± 0.0^b^	2.28 ± 0.48^c^	1.14 ± 0.37^d.e^
Edema	0.0 ± 0.0	3.71 ± 0.48^a^	1.85 ± 0.69^c^	1.14 ± 0.37^c.f^

^a^ *p* < 0.01 as compared to group 1, ^b^ *p* < 0.001 as compared to group 1, ^c^ *p* < 0.01 as compared to group 2, ^d^ *p* < 0.001 as compared to group 2, ^e^ *p* < 0.01 as compared to group 3, and ^f^ *p* < 0.05 as compared to group 3.
